# The “Hygiene Hypothesis” and the Lessons Learnt From Farm Studies

**DOI:** 10.3389/fimmu.2021.635522

**Published:** 2021-03-18

**Authors:** Erika von Mutius

**Affiliations:** ^1^ Dr. von Hauner Children’s Hospital, University Hospital, LMU Munich, Munich, Germany; ^2^ Helmholtz Zentrum Muenchen—German Research Center for Environmental Health, Institute of Asthma and Allergy Prevention, Munich, Germany; ^3^ Comprehensive Pneumology Center Munich (CPC-M), Member of the German Center for Lung Research, Munich, Germany

**Keywords:** asthma, children, allergy, immunology, epidemiology

We celebrate the 30th anniversary of the “hygiene hypothesis”, which has been a cornerstone for research into asthma and allergic diseases for many of us. It appeared while we witnessed the rapid increase of these conditions in the westernized world by the end of last century (1). It has stimulated thought of many researchers resulting in numerous more or less modified hypotheses meandering in diverse gestalt through the scientific landscape. It all started with an epidemiological observation about significantly decreased risk of allergic sensitization and hay fever in subjects having many siblings. This observation was counterintuitive at that time when the prevalent paradigm stated that viral infections cause asthma. However, the observation was confirmed many times in independent populations and is one of the most robust epidemiological findings in the context of allergy (2). Over the years the epidemiological gestalt changed from siblings to day care, oro-fecal and other infections and then to farm exposures. Interestingly, the farm effect is independent of the “sibling effect” (3). The gestalt also took on various immunological garments from a Th1-Th2 dichotomy to regulatory networks. Lately, the technological progress allowing exploration of the world of microbiomes has revitalized the debates around the “hygiene hypothesis” with tantalizing findings from mouse experiments and population-based studies.

The “hygiene hypothesis” has also been a cornerstone of my scientific life resulting in my continued interest in the farm populations that I have been following with many colleagues since the beginning of this century. In this modified gestalt, the farm exposures may be considered strong support for a hypothesis that may be rephrased as pointing to the importance of microbial exposures for the development of childhood asthma and allergies. The concept is intuitively easily understandable which has resulted in a widespread perception by the lay press. Yet, we still wrestle with the identification and mechanistic understanding of the relevant building blocks that may allow translation into prevention of childhood asthma and allergies. In the following, I attempt to distil some lessons from the farm studies.

The protective effect of a traditional farm exposure on the development of childhood asthma and allergies as documented in numerous studies is very robust. Similar to the allergy protective “sibling effect” and the asthma risk by exposure to moulds and active/passive smoking it is a remarkably reproducible finding across populations and continents. Moreover, the effects are strong. The most consistent finding, which relates to allergic sensitization and hay fever, shows odds ratios around 0.5 suggesting halving of risk (4). Findings for asthma seem somewhat weaker and less reproducible which may be attributable to the many facets of the asthma syndrome. These observations may suggest a strong extrinsic factor that once identified could serve as novel prevention strategy for these illnesses.

We have identified two main pillars of the protective farm effect, one being the exposure to animal sheds, in particular cowsheds and the second the consumption of unprocessed cow’s milk (5). It is tempting to speculate that one unifying exposure may underlie these seemingly distinct exposures. The working group of Erika Jensen-Jarolim proposes that ß-lactoglobulin, which is found in cow’s milk and urine and thus also in ambient air of cowsheds, carries farm-specific ligands which render this lipocalin tolerogenic rather than allergenic (6). Thereby ß-lactoglobulin could act as important transport protein presenting its allergy- and potentially asthma-protective cargo to competent immune cells. This concept awaits however, confirmation in mouse studies of experimental allergic asthma and allergy. If substantiated the nature of the cargo needs further investigation and the relative contribution of the transporter *versus* the cargo (and the diversity of the cargo) must be resolved. It seems conceivable that the farm environment confers not only the protective exposures, but also the transporters that enhance the protective effects by optimizing presentation to competent immune cells.

From the epidemiological observations, diversity of exposures in the farm environment has however been a central theme. We have so far not found one single component conferring protection. One must bear in mind that cowsheds and unprocessed cow’s milk are “soups” containing myriads of potentially relevant elements. We have shown that an increased diversity of food introduction protects from food allergy, atopic dermatitis and asthma (7). Moreover, the diversity of farm animal exposure during pregnancy has been associated with lower risk of atopic dermatitis and higher IFN-y and TNF-α levels in supernatants of cord blood mononuclear cells stimulated with LPS (8, 9). Finally, the diversity of the environmental and human nasal microbiome, respectively, have been associated with lower risk of asthma in the farm populations (10).

A strong signal with diversity results in a low likelihood to find the one “magic bullet” explaining the protective associations. It can in turn be interpreted as a multitude of additive (weak) effects interacting with a multitude of host factors in the general population which is made up of subjects with very diverse genetic and immune response backgrounds. Alternatively, diversity may harbour a limited number of relevant, necessary and sufficient elements or hubs in exposure networks which drive the protective effects. We have some evidence that these necessary elements exist because in experimental studies of farm exposure, i.e. extracts from cowshed dust extracts do no longer protect mice from allergic asthma when they are devoid of MYD88/TRIF and epithelial A20 signalling (11, 12). Thereby, innate immune responses may be essential elements for the protective effect, but the precise nature of these elements stills awaits elucidation.

The complexity of the interplay of protective elements may be further increased by the multitude of exposure routes that may matter. Environmental exposures such as the indoor microbiome or the stay in cowsheds may be inhaled or ingested. In fact, we have seen that both the nasal and gut microbiome are influenced by these external exposures because young children breathe in airborne matter and put their contaminated fingers in their mouth thereby ingesting external compounds. In addition, ingestion of relevant exposures such as unprocessed cow’s milk or a diverse introduction of solid foods further shapes the gut microbiome and its development (13). We have unfortunately not investigated skin exposures, but these may also add to the complex interplay of farm exposure routes.

We are therefore left with the impression that key elements of protection within the farm environment affect a number of body compartments (upper and lower airways, gut and skin) in probably redundant and overlapping pathways, which may in turn confer protection for a large majority of exposed subjects with rather diverse genetic and immune response backgrounds.

If this notion was correct, then translation into novel asthma preventive approaches will have to be multifaceted. As discussed in a recent review (14), it seems unlikely that asthma phenotypes are distinct conditions with distinct underlying pathologies resulting in exclusive unambiguous disease categories. Complex diseases such as asthma are more likely to consist of combinations of various traits and underlying redundant mechanisms given the many weak genetic and environmental effects and their interactions on disease development. In such a scenario the one and only causal mechanism can neither be found nor successfully targeted for prevention. Then only some facets of the disease will be addressed which however results in weak effects on a population level. In a complex disease a combination of a multitude of involved mechanisms matters which should preferably all be targeted if all of asthma should be prevented. Given that the “farm effect” on asthma is strong across multiple populations it seems more likely that the multitude and diversity of exposures across several routes of exposures (upper and lower airways, gut, skin) contributes to the overall protective effect.

## Author Contributions

The author confirms being the sole contributor of this work and has approved it for publication.

## Conflict of Interest

Personal fees from Pharmaventures, OM Pharma S. A., Peptinnovate Ltd., Böhringer Ingelheim International GmbH.

Patent LU101064: Barn dust extract for the prevention and treatment of diseases pending,

Patent EP2361632: Specific environmental bacteria for the protection from and/or the treatment of allergic, chronic inflammatory and/or autoimmune disorders with royalties paid to ProtectImmun GmbH,

Patent EP 1411977: Composition containing bacterial antigens used for the prophylaxis and the treatment of allergic diseases. licensed to ProtectImmun GmbH,

Patent EP1637147: Stable dust extract for allergy protection licensed to ProtectImmun GmbH,

Patent EP 1964570: Pharmaceutical compound to protect against allergies and inflammatory diseases licensed to ProtectImmun GmbH.
